# A Potent Postentry Restriction to Primate Lentiviruses in a Yinpterochiropteran Bat

**DOI:** 10.1128/mBio.01854-20

**Published:** 2020-09-15

**Authors:** James H. Morrison, Caitlin Miller, Laura Bankers, Gary Crameri, Lin-Fa Wang, Eric M. Poeschla

**Affiliations:** a Division of Infectious Diseases, University of Colorado, Aurora, Colorado, USA; b CSIRO Australian Animal Health Laboratory, Geelong, Australia; c Programme in Emerging Infectious Diseases, Duke-NUS Medical School, Singapore; Rutgers—Robert Wood Johnson Medical School

**Keywords:** retrovirus, HIV-1, lentivirus, bats, restriction factors, cyclophilin A, MX2, Megachiroptera, Yinpterchiroptera, *Pteropus alecto*, restriction factor

## Abstract

Bats are primary reservoirs for multiple lethal human viruses, such as Ebola, Nipah, Hendra, rabies, severe acute respiratory syndrome coronavirus (SARS-CoV), Middle East respiratory syndrome-related coronavirus (MERS-CoV), and, most recently, SARS-CoV-2. The innate immune systems of these immensely abundant, anciently diverged mammals remain insufficiently characterized. While bat genomes contain many endogenous retroviral elements indicative of past exogenous infections, little is known about restrictions to extant retroviruses. Here, we describe a major postentry restriction in cells of the yinpterochiropteran bat Pteropus alecto. Primate lentiviruses (HIV-1, SIVmac) were potently blocked at early life cycle steps, with up to 1,000-fold decreases in infectivity. The block was specific, because nonprimate lentiviruses such as equine infectious anemia virus and feline immunodeficiency virus were unimpaired, as were foamy retroviruses. Interspecies heterokaryons demonstrated a dominant block consistent with restriction of incoming viruses. Several features suggested potential TRIM5 (tripartite motif 5) or myxovirus resistance protein 2 (MX2) protein restriction, including postentry action, cyclosporine sensitivity, and reversal by capsid cyclophilin A (CypA) binding loop mutations. Viral nuclear import was significantly reduced, and this deficit was substantially rescued by cyclosporine treatment. However, saturation with HIV-1 virus-like particles did not relieve the restriction at all. P. alecto TRIM5 was inactive against HIV-1 although it blocked the gammaretrovirus N-tropic murine leukemia virus. Despite major divergence in a critical N-terminal motif required for human MX2 activity, P. alecto MX2 had anti-HIV activity. However, this did not quantitatively account for the restriction and was independent of and synergistic with an additional CypA-dependent restriction. These results reveal a novel, specific restriction to primate lentiviruses in the Pteropodidae and advance understanding of bat innate immunity.

## Abstract (precis)

The COVID-19 pandemic suggests that bat innate immune systems are insufficiently characterized relative to the medical importance of these animals. Retroviruses, e.g., HIV-1, can be severe pathogens when they cross species barriers, and bat restrictions corresponding to retroviruses are comparatively unstudied. Here, we compared the abilities of retroviruses from three genera (*Lentivirus*, *Gammaretrovirus*, and *Spumavirus*) to infect cells of the large fruit-eating bat P. alecto and other mammals. We identified a major, specific postentry restriction to primate lentiviruses. HIV-1 and SIVmac are potently blocked at early life cycle steps, but nonprimate lentiviruses and foamy retroviruses are entirely unrestricted. Despite acting postentry and in a CypA-dependent manner with features reminiscent of antiretroviral factors from other mammals, this restriction was not saturable with virus-like particles and was independent of P. alecto TRIM5, TRIM21, TRIM22, TRIM34, and MX2. These results identify a novel restriction and highlight cyclophilin-capsid interactions as ancient species-specific determinants of retroviral infection.

## INTRODUCTION

Cell-autonomous antiviral restriction factors, which act dominantly to inhibit replication of viruses, are important barriers to cross-species transmission. For retroviruses, proteins such as Fv1, APOBEC, tripartite motif 5α (TRIM5α), TRIMCyp, myxovirus resistance protein 2 (MX2 [MXB]), and tetherin pose barriers to host switching and exert strong selection on viruses when this occurs (1).

The mammalian order Chiroptera contains about 20% of all mammalian species (more than 1,200), making it the second most species-rich mammalian order besides Rodentia (2, 3). Bats are proven or strongly implicated reservoir hosts for diverse emerging viruses responsible for severe, high-lethality outbreaks in humans and apes, including filoviruses (Ebolavirus, Marburg virus), paramyxoviruses (Hendra virus, Nipah virus), the neuropathogenic lyssaviruses (e.g., rabies virus), and coronaviruses (severe acute respiratory syndrome coronavirus [SARS-CoV], Middle East respiratory syndrome-related coronavirus [MERS-CoV], and SARS-CoV-2) (4–6). With exceptions such as rabies virus, bats can carry human-pathogenic viruses without apparent disease or inflammation (7). The prominence of bats in this regard and the cellular mechanisms that determine the special properties of viral susceptibility, cell- and species-level restriction, unusual tolerance to chronic carriage, asymptomatic shedding, and spillover potential into other mammals are poorly understood. The complex issue of whether bats have evolved to be especially convivial viral reservoirs has drawn attention and has been reviewed well elsewhere (3, 7–9). Some analyses (2, 10) but not others (11) have suggested that, per species, bats host more zoonotic viruses than rodents and other mammalian orders. Bat characteristics that could contribute to disproportionate viral reservoir capacity include abundance; aggregation in large, dense colonies; ability to propagate viruses widely as the only mammals that have achieved flight; extreme longevity relative to size and metabolic rate (many species live longer than 25 years) (7); and distinctive innate immune system properties (12–14).

Available evidence suggests that the Chiroptera diverged from other placental mammals of the superorder Laurasiatheria in the late Cretaceous era, about 80 to 90 million years ago (7, 15), and hence comprise a relatively ancient order. It has been speculated that the genetic arms race between bats and viruses may have reached, in relative terms, a state of equilibrium (3, 7). However, at the cellular level, little is known, as the innate immune systems of bats have received little specific study and studies of their adaptive immune systems are also few in number. Recent reports have suggested unique innate immune system features, such as a contracted interferon (IFN) gene repertoire; different IFN expression patterns; dampened NLRP3 inflammasome activity; and reduced signaling through STING, a key adaptor protein in DNA sensing pathways (12–14, 16). A mutation of a highly conserved serine in bat STING proteins attenuates the IFN response to cytoplasmic DNA (14), which may be an adaptation to increased DNA damage generated by the metabolic demands of flight (17). Other yet-to-be-elucidated pathways may also exist in bats as part of their evolution to flight (18).

The Chiroptera were traditionally classified into two main suborders, the Megachiroptera (large, Old World fruit bats) and the Microchiroptera (small, echolocating, insectivorous bats). Based on molecular phylogenetic evidence, two alternative suborders have also been proposed (19). The Yangochiroptera suborder contains 14 families, all echolocating. The Yinpterochiroptera suborder is comprised of six families that include the Pteropodidae (nonecholocating megabats) and the Rhinolophidae (small, echolocating bats implicated as reservoirs for SARS viruses). The Pteropodidae are Old World fruit bats that inhabit an immense geographic range, from eastern Africa to Oceania and the Polynesian archipelago (20). Pteropid species such as Pteropus alecto and P. vampyrus, which are native to regions of Oceania, are principal reservoirs for the zoonotic paramyxoviruses Hendra virus and Nipah virus (21, 22). Fruit bats also harbor influenza A viruses with human zoonotic potential; remarkably, while all other influenza A viruses enter cells via sialic acid receptors, bat influenza viruses instead use major histocompatibility complex class II (MHC-I) receptors and can utilize the MHC-II molecules of humans, other mammals, and birds (23, 24).

Retroviruses are RNA viruses that perform reverse transcription of their plus-sense RNA genomes and insert the resulting double-stranded cDNA into a host chromosome. Bat genomes contain endogenous retroviruses (ERVs) from the *Betaretrovirus*, *Gammaretrovirus*, and *Deltaretrovirus* genera (25–28), and an exogenous gammaretrovirus has also been identified (29). Germline endogenization represents the outcome of presumably very rare integrations into gametes or gamete progenitor cells. The abundance and variety of bat ERVs imply extensive prior exogenous retroviral colonization and suggest not only that bats have been hosts to retroviruses of these genera through substantial spans of their evolutionary history but also that cross-species transmissions with other mammals and marsupials have been common (26). Here, we studied the abilities of multiple retroviruses spanning three retroviral genera (*Lentivirus*, *Gammaretrovirus*, and *Spumavirus*) to infect cells from P. alecto and other mammals and identify the first target cell-dependent retroviral restriction in bats. We cloned and analyzed phylogenetic relationships and antiviral activities of multiple P. alecto restriction factor genes encoding TRIM5 (tripartite motif 5), TRIM21, TRIM22, TRIM34, and MX2. We also used virus-specific and species-specific infection patterns and subsequent molecular analyses to identify and characterize species- and virus-specific restrictions.

## RESULTS

### Primate lentiviral species-specific restriction in Pteropus alecto cells.

**FIG 1:**
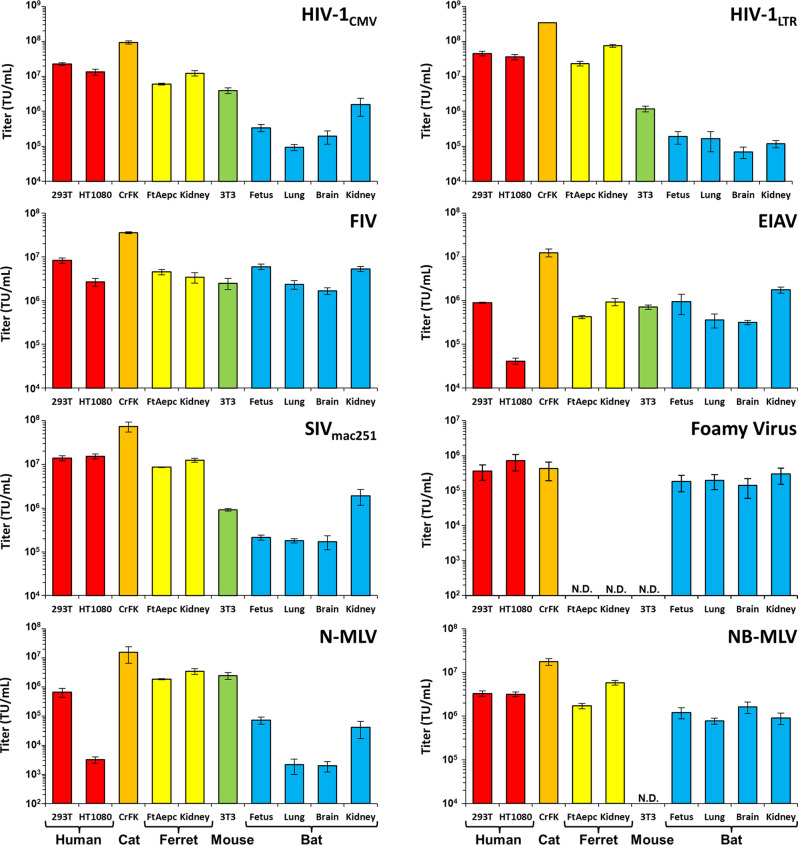
Pteropus alecto cells restrict primate lentiviruses. Cells from diverse mammalian species were infected with the indicated single-round retroviral vectors encoding eGFP in 3-fold dilutions over a 3-log range of viral inputs. At 72 h postinfection, cells were analyzed for eGFP expression and the percentage of GFP-positive cells was used to calculate the number of transducing units (TU) per milliliter of vector for each cell line. Bars represent the average titer ± standard deviation. N.D., not determined. This experiment was repeated at least 3 times for each virus with the same results. To overcome the lack of compatible viral receptors on bat cells, and to enable comparable viral attachment and entry results, vesicular stomatitis virus glycoprotein G (VSV-G) was used to pseudotype viral vectors, with the exception of foamy virus, which does not require pseudotyping in order to infect diverse vertebrate cell lines. Additionally, to standardize transcriptional activity and enable direct comparison of viral infectivities, the vectors employed an internal CMV promoter that mediates eGFP expression. For HIV-1, a nearly full-length HIV-1 NL4-3-derived *env*-negative reporter virus (HIV-1_LTR_) that expresses eGFP from the *nef* open reading frame under HIV-1 U3 and Tat control (70) was also tested.

We first tested the intrinsic susceptibility to infection by diverse retroviruses in four cell lines that were derived from P. alecto brain, lung, kidney, and whole-fetus tissue, respectively (30). Single-cycle viral vectors derived from three retroviral genera—gammaretroviridae (NB-tropic murine leukemia virus [NB-MLV]), spumaviridae (foamy virus), and lentiviridae (feline immunodeficiency virus [FIV] and equine infectious anemia virus [EIAV])—were able to infect Pteropus alecto kidney, lung, brain, and fetus cell lines with efficiency comparable to that seen with well-established human, feline, ferret, and mouse cell lines (Fig. 1; see also Fig. S1 and S2 in the supplemental material).

10.1128/mBio.01854-20.1 FIG S1 Limiting-dilution retrovirus infections by cell line. Titers were predetermined for vectors on 293T cells for all experiments represented in the 10 panels in Fig. S1 (panels A to J); hence, MOI is based on the 293T cell titer. The HIV-1 vector “HIV-1 CMV” is a minimal 3-plasmid system with an internal CMV promoter, while HIV-1 LTR is a reporter virus that has a frame-shifting *env* deletion, a truncated *vpr* gene, and *egfp* replacing *nef*. Download FIG S1, TIF file, 2.8 MB. Copyright © 2020 Morrison et al. 2020 Morrison et al. This content is distributed under the terms of the Creative Commons Attribution 4.0 International license.

10.1128/mBio.01854-20.2 FIG S2 Limiting-dilution retrovirus infections by virus. A single vector preparation for each virus was serially diluted on each cell line for all experiments represented in the 7 panels in Fig. S2 (panels A to G). Cell lines are color coded by species as follows: red, human; orange/yellow, cat; brown, ferret; blue or purple, bat. Download FIG S2, TIF file, 2.9 MB. Copyright © 2020 Morrison et al. 2020 Morrison et al. This content is distributed under the terms of the Creative Commons Attribution 4.0 International license.

In interesting contrast to the nonprimate lentiviruses, the primate lentiviruses HIV-1 and simian [macaque] immunodeficiency virus 251 (SIV_mac251_) were severely impaired in pteropid bat cell lines, with over a 100-fold decrease in viral titers observed compared to other mammalian cell lines (Fig. 1; see also Fig. S1 and S2). N-tropic murine leukemia virus (N-MLV) is identical to NB-MLV except for a capsid mutation at residue 110 that confers sensitivity of N-MLV to the murine *Fv1^b^* allele and TRIM5α (31). Whereas NB-MLV had similar titers in bat cells versus human, cat, and ferret cell lines, N-MLV displayed reduced infectivity in the P. alecto cell lines relative to other mammalian species cells (as well as in HT1080 cells as expected), suggesting that a capsid-targeting resistance factor could exist in P. alecto cells.

As indicated in Table S1 in the supplemental material, we aggregated the titer data for the primate viruses (HIV-1_CMV_ [where “CMV” represents a minimal 3-plasmid system with an internal cytomegalovirus {CMV} promoter], HIV-1_LTR_ [HIV with a long terminal repeat], and SIV_mac_), for the two nonprimate lentiviruses (FIV and EIAV), and for the foamy virus to calculate the ratio of mean titers in 293T, HT1080, and Crandell-Rees feline kidney (CrFK) cells to mean titers from all four of the P. alecto lines. HIV-1_LTR_ (which has a full-length genome except for a partial deletion of *env* and a replacement of *nef* by *gfp*) displayed a further reduction in enhanced green fluorescent protein (eGFP) expression compared to HIV-1_CMV_, suggesting that early gene expression, which is under HIV-1 Tat transcriptional control, is further attenuated in the bat cells. Nevertheless, HIV-1_CMV_ is clearly restricted postentry compared to the analogous internally human cytomegalovirus (hCMV)-promoted FIV and EIAV vectors (Table S1) (Fig. 1; see also Fig. S1 and S2).

10.1128/mBio.01854-20.6 TABLE S1 Ratio of titer in indicated cell line to mean titer in the four Pteropus alecto lines. Ratios were calculated from the titer data shown in Fig. 1. Rows 293T, HT1080, and CrFK show the titer of the virus from each column in the indicated cell line divided by the mean titer of the four Pteropus alecto cell lines. The “Mean ratio” row shows averages of the 293T, HT1080, and CrFK titer ratios for each virus. Download Table S1, TIF file, 2.7 MB. Copyright © 2020 Morrison et al. 2020 Morrison et al. This content is distributed under the terms of the Creative Commons Attribution 4.0 International license.

### The restriction to HIV-1 is dominant but not saturable.

**FIG 2:**
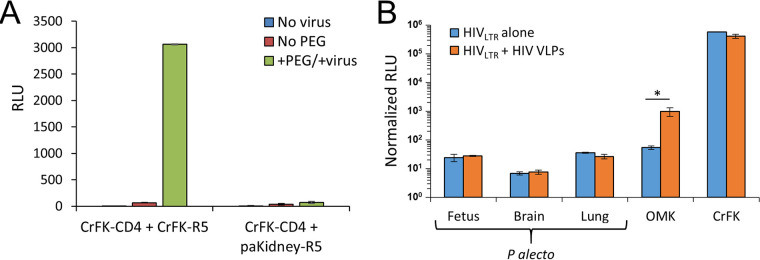
The block to HIV infection in P. alecto cell is dominant but not saturable. (A) Heterokaryon infection. The indicated cells were cocultured for 24 h prior to fusion performed with polyethylene glycol (PEG 500) or control treatment with PBS. Levels of fusion efficiency were verified to be equivalent by counting multinucleated cells. At 24 h postfusion, cells were infected with R5 HIV-1, and after an additional 48 h, cells were counted and analyzed for luminescence. The experiment was repeated four times with the same results. (B) Inability of VLPs to abrogate the block in P. alecto cells. Cells were first infected with VLPs (VSV-G-pseudotyped HIV-1 particles with no viral genome). At 30 min later, the cells were infected with HIV-1_LTR_-luc(VSV-G). Four days after infection, the cells were counted and analyzed for luminescence. Error bars represent the standard deviations of luminescence data calculated in triplicate. This experiment was repeated three times with the same results. *, *P* < 0.01.

The virus- and species-specific infection patterns that we observed are consistent with either the absence of a viral dependency factor(s) or the presence of one or more antiviral restriction factors in P. alecto cells. To distinguish these possibilities, we generated heterokaryons between the P. alecto kidney cell line (paKiT01) and the permissive feline kidney cell line CrFK and infected them with HIV_CMV_ produced with a native CCR5 (R5)-tropic Env glycoprotein. To strictly limit infection to interspecies heterokaryons, the CrFK cells were first engineered to stably express human CD4 and the paKiT01 cells to express human CCR5 (32). Only fused cells that have both CD4 and CCR5 can support entry of R5-tropic HIV-1. While CrFK-CD4/CrFK-CCR5 heterokaryons supported infection, no luciferase activity was detected in CrFK-CD4/paKiT01-CCR5 heterokaryons (Fig. 2A). This result is consistent with a dominant block in the P. alecto cells rather than lack of an HIV-1-specific dependency factor.

Next, we tested whether the block to HIV-1 infectivity could be relieved by virus-like particle (VLP) saturation, which is a well-demonstrated feature of TRIM5α and TRIMCyp restriction of retroviral infection (33, 34). Vesicular stomatitis virus G (VSV G)-pseudotyped HIV-1 VLPs containing no viral genome were utilized to saturate cells shortly before they were infected with an HIV-1 luciferase vector. TRIMCyp-mediated restriction of HIV-1 in owl monkey kidney (OMK) cells was significantly reduced by VLP saturation (18.2-fold increase in infectivity), whereas treatment of feline CrFK cells, which lack endogenous TRIM5 or TRIMCyp proteins (35), resulted in no change in reporter virus infectivity (Fig. 2B). Bat fetus, brain, and lung cells showed no change in HIV-1 infectivity with VLP saturation (Fig. 2B).

### Disruption of cyclophilin A (CypA) interaction with HIV-1 capsid relieves the block to infectivity.

As noted above, N-MLV, which is sensitive to the murine *Fv1^b^* allele, was restricted in bat cells whereas NB-MLV was not. However, the distribution of *Fv1* in *Muroidea* indicates that it was inserted approximately 45 to 55 million years ago, which dates to a time point after the divergence of *Chiroptera* from *Rodentia* over 70 million years ago (36). We found no open reading frames with homology to *Fv1* when we used BLASTN and tBlastn queries to search the P. alecto genome and transcriptome (8, 17).

Known early-acting lentivirus-targeted restriction factors include APOBEC3 (A3) family cytidine deaminases (37), TRIM5α (33), TRIMCyp (34), SAMHD1 (38, 39), and MX2 (40–43). The P. alecto A3 locus was recently investigated, and four of the proteins were found to inhibit HIV-1 infectivity (44). However, as the antiretroviral activities of these and other A3 proteins require viral particle incorporation in virus-producing cells (37), A3-related effects could be excluded in the postentry restriction as all of our viral stocks were produced in 293T cells.

**FIG 3:**
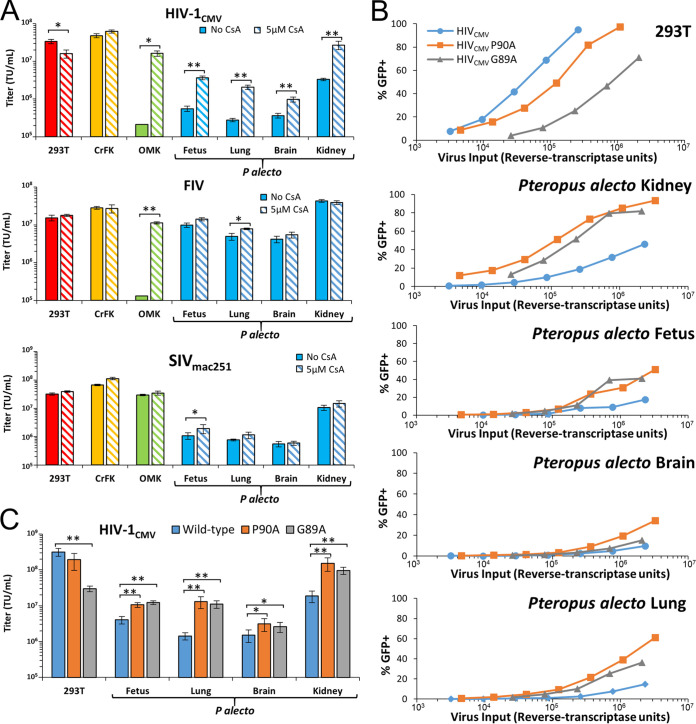
Inhibition of cyclophilin A binding relieves restriction in bat cells. (A) The indicated cell lines were infected with vectors encoding eGFP in 3-fold dilutions in the presence of either 5 μM CsA or DMSO. At 72 h postinfection, cells were analyzed for eGFP expression, and the percentage of eGFP-positive cells was used to calculate the number of transducing units (TU) per milliliter of vector for each cell line. *, *P* < 0.01; **, *P* < 0.001. (B and C) The indicated cells were infected and analyzed as described above. HIV-1_CMV_ vectors harboring a wild-type capsid or a capsid with mutations P90A and G89A were used. Percent eGFP data were calculated (B) and used to determine the average titer of each vector on each cell line (*, *P* < 0.05; **, *P* < 0.01) (C). These experiments were repeated at least 2 times each.

Genomic analyses have revealed the presence of P. alecto TRIM5 and myxovirus resistance (MX1 and MX2/MxB) orthologues (17). We reasoned that the nature of the block to primate lentiviral infection could be determined indirectly with a series of viral challenges under specific conditions previously demonstrated to be prominent features of TRIM5 or MX2 restriction. To investigate putative TRIMCyp or MX2 restriction activities, viral challenges were first repeated in the presence of cyclosporine (CsA), an immunosuppressant drug that relieves TRIMCyp and MX2 restriction by preventing postentry binding of the peptidylprolyl isomerase cyclophilin A (CypA) to the viral capsid core, which is necessary for viral restriction mediated by either of these factors (33, 40–43). As has been reported previously (34), CsA markedly enhanced infection by HIV-1 and FIV in owl monkey kidney (OMK) cells, which express high levels of TRIMCyp protein (Fig. 3A). CsA treatment resulted in a 2.1-fold decrease in HIV-1 infectivity in human 293T cells but caused 6.6-, 7.4-, 3.7-, and 8.2-fold increases in HIV-1 infection of P. alecto fetus, lung, brain, and kidney cell lines, respectively (Fig. 3A). In contrast, FIV and SIV_mac251_ infections in bat cells were generally unperturbed by CsA treatment (Fig. 3A), though CsA treatment improved FIV infectivity in the bat lung cells (1.6-fold) and SIV infectivity in the bat fetus cells (1.8-fold). These key data demonstrate that the P. alecto restriction to HIV-1 infection is partially controlled through a CypA-dependent mechanism, whereas the restriction to SIV_mac251_ infection is almost entirely independent of CypA.

The influence of CypA on HIV-1 infection is complex, with reported effects on capsid stability, nuclear import of viral DNA, reverse transcription, and shielding of viral nucleic acids from host sensor/effector systems such as TRIM5α (45–50). To confirm our results, CypA capsid binding mutants were tested for infectivity in human and bat cell lines. As we observed with CsA treatment, HIV-1 produced with either of the well-characterized P90A or G89A capsid mutants in the CypA binding loop had reduced infectivity per reverse transcriptase unit in 293T cells (1.7-fold and 10.4-fold for P90A and G89A, respectively). However, the opposite pattern was observed in bat cells, where both mutants had increased infectivity (Fig. 3B and C). While HIV-1 with a wild-type (WT) capsid had 51-fold increased infectivity in 293T cells compared to the mean titer in the four bat cell lines, the difference for P90A was only 2.9-fold and G89A had equivalent titers in 293T cells and bat cells (Fig. 3C). These results confirm that the postentry restriction to HIV-1 in pteropid cells is strongly dependent on interaction of the viral capsid protein with cellular cyclophilin A or cyclophilin A domain-containing proteins.

### P. alecto tripartite motif proteins account for N-MLV but not HIV-1 restriction.

We focused next on the involvement of the tripartite motif-containing (TRIM) proteins, in particular, TRIM5 and TRIMCyp. The ability of these proteins to recognize and restrict incoming retroviral particles is well documented in a number of diverse mammalian species, including primates and bovids (33, 51). TRIM proteins are functionally modular, with a viral capsid recognition domain (SPRY or CYP) and effector domains (RING, B-box, and coiled-coil) that enable multimerization, direct viral disruption, and mediate intracellular signaling following recognition of a susceptible retroviral capsid core (33, 34, 52). Searches for a TRIMCyp protein orthologue in the P. alecto genome and transcriptome yielded no candidates. However, a putative P. alecto TRIM5 orthologue with 78% coverage and 57% identity to human TRIM5α (GenBank accession no. ELK09387.1) was previously annotated during a high-throughput analysis of multiple bat genomes (17). We were unable to directly amplify a cDNA for the full-length open reading frame corresponding to this protein from any of the P. alecto cell lines. We were, however, able to isolate a fragment corresponding to its C-terminal SPRY domain from the kidney and lung cell line cDNA, which was then used in 5′ rapid amplification of cDNA ends (5′RACE). The RACE experiment yielded transcripts corresponding to a P. alecto TRIM5 protein with 100% coverage and 62% identity to human TRIM5α from both cell lines. The 5′RACE-derived TRIM5 transcript (Genbank accession no. MT649092) was aligned with the putative TRIM5 orthologue ELK09387.1. The two differ substantially at the N terminus, with the 5′RACE-derived sequence encoding about 100 additional amino acids (Fig. S3). The 5′RACE transcripts contained in-frame stop codons upstream of the initiator methionine, demonstrating that this sequence likely reflects the longest expressed isoform. We were not able to identify any alternative isoforms for TRIM5 and were unable to isolate TRIM5 transcripts encoding the N-terminal region of ELK09387.1. We conclude that the P. alecto TRIM5 transcript that we isolated is the predominantly expressed form and that ELK09387.1 may be incorrectly annotated. cDNAs for three additional P. alecto TRIM proteins were similarly cloned and are described below.

10.1128/mBio.01854-20.3 FIG S3 5′RACE-derived Pteropus alecto TRIM5 (Genbank accession no. MT649092) alignment with putative sequence. 5′RACE was performed on RNA derived from Pteropus alecto kidney and lung cells using primers anchored in the 5′ region of the gene (SPRY). Transcripts identified by 5′RACE were used to clone a full-length TRIM5 cDNA (denoted CDNA at the top), and the sequence was aligned in Clustal Omega to the putative Pteropus alecto TRIM5 ELK09387.1 sequence. Download FIG S3, TIF file, 2.4 MB. Copyright © 2020 Morrison et al. 2020 Morrison et al. This content is distributed under the terms of the Creative Commons Attribution 4.0 International license.

**FIG 4:**
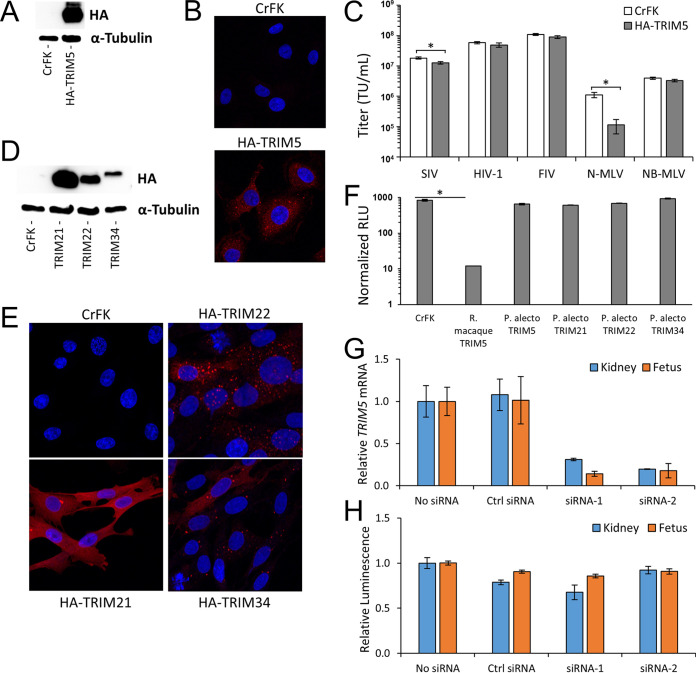
Pteropus alecto TRIM proteins do not restrict HIV-1 activity. (A to C) CrFK cells were transduced with lentiviral vector encoding HA epitope-tagged Pteropus alecto TRIM5 and selected with puromycin. (A) Stable TRIM5 expression was verified by immunoblotting for HA and α-tubulin. (B) Indirect immunofluorescence against the HA epitope was performed (blue, DAPI; red, anti-HA). Wild-type and TRIM5-expressing CrFK cells were infected with limiting dilutions of the indicated eGFP-expressing single-cycle retroviral vectors. (C) At 48 h postinfection, eGFP expression was quantified by flow cytometry and the percentages of GFP-positive cells were used to determine the infectious titer (transducing units per milliliter) for each vector on wild-type or TRIM5-expressing cells. (D and E) Immunoblotting (D) and indirect immunofluorescence (E) were performed as described for panels A and B with CrFK cells expressing additional HA epitope-tagged Pteropus alecto TRIM proteins. (F) Wild-type and stable TRIM-expressing CrFK cells were infected with HIV-1_LTR_ Luc, and 72 h postinfection, cells were counted, lysed, and analyzed for luminescence. Relative light unit (RLU) data were normalized to the number of healthy cells and are shown as the average RLU reading ± standard deviation. (G and H) P. alecto kidney or fetus cells were transfected with *TRIM5*-specific siRNA 48 h prior to infection with a luciferase-encoding HIV-1 strain. *TRIM5* mRNA expression (G) and virally encoded luciferase activity (H) were measured 48 h following viral infection. Luciferase activity was normalized to untreated cells. *, *P* < 0.01.

Having identified and cloned the full-length P. alecto TRIM5 cDNA, we used a lentiviral vector to stably express the predicted protein with an N-terminally incorporated hemagglutinin (HA) epitope tag in CrFK cells (Fig. 4A). CrFK cells were used because they lack endogenous TRIM5 and other known postentry restrictions but support retroviral restriction when TRIM5 proteins derived from other species are ectopically expressed (35, 53). P. alecto TRIM5 had a punctate intracellular distribution with visible cytoplasmic bodies that resembled those that are well described (54) for human TRIM5α (Fig. 4B). Expression of TRIM5 in CrFK cells resulted in a 10-fold decrease in infectivity of N-MLV but had no effect on infectivity of NB-MLV, HIV-1, or FIV (Fig. 4C). SIV infectivity was slightly impaired in the TRIM5-expressing cells (1.4-fold decrease). These results indicate that P. alecto TRIM5 has antiviral activity against the gammaretroviral genus but has weak or no recognition of the lentiviruses tested. Furthermore, this suggests the restriction to N-MLV observed in the P. alecto cell lines (Fig. 1) stems from TRIM5 expression in those cells.

Human and mouse genomes harbor 65 and 64 tripartite motif (TRIM)-containing genes, respectively, and many are increasingly recognized as important factors in innate immunity (55). To test the possibility that other TRIM family proteins gained retroviral targeting in pteropid bat species, we cloned cDNAs for and determined the antiviral capabilities of three additional TRIM family proteins: TRIM21, TRIM22, and TRIM34. These three were chosen because we were able to identify clear homologs in the P. alecto genome and because all three contain a C-terminal SPRY domain, which is a protein-protein interaction module that is responsible for direct interactions with retroviral capsid cores (56–58). TRIM34 was also recently shown to restrict HIV-1 through a capsid sequence-dependent mechanism (59). We used multiple-sequence alignments of known TRIM orthologues and our P. alecto TRIM sequences to generate a phylogenetic tree, which verified that each pteropid gene is appropriately named and that orthologous TRIM proteins cluster together phylogenetically (Fig. S4). Following stable expression of each TRIM protein (Fig. 4D), subcellular localization of each factor was determined by indirect immunofluorescence. P. alecto TRIM21 was diffusely distributed in the cytoplasm, whereas the majority of TRIM22 and TRIM34 proteins formed apparent cytoplasmic bodies, with a minority of the proteins locating in a more diffuse pattern (Fig. 4E). The location of these bat proteins is congruent with prior reports for the human versions (59).

10.1128/mBio.01854-20.4 FIG S4 Phylogenetic tree of SPRY-containing Pteropus alecto TRIM family proteins with mammalian orthologues. cDNAs corresponding to the denoted Pteropus alecto TRIM genes were isolated from cDNA from the kidney or lung cells, and each entire open-reading frame was sequenced. A multiple-sequence alignment was then generated in Clustal Omega. This alignment was used to generate a maximum likelihood phylogenetic tree using the JTT matrix-based model, as implemented in MEGA7. The tree with the highest log likelihood is shown. Branch values represent bootstrap percentages determined after 1,000 replicates were analyzed. Download FIG S4, TIF file, 2.8 MB. Copyright © 2020 Morrison et al. 2020 Morrison et al. This content is distributed under the terms of the Creative Commons Attribution 4.0 International license.

However, in contrast to the highly restrictive TRIM5α protein from rhesus macaques, none of these P. alecto homologs displayed antiviral activity against HIV-1 (Fig. 4F). Finally, by knocking down TRIM5 prior to infection, we confirmed that endogenous TRIM5 protein in the P. alecto cells did not contribute to the restriction of HIV-1. Despite efficient knockdown of the TRIM5 mRNA (Fig. 4G), there was not a significant difference in HIV-1 infectivity (Fig. 4H). These results demonstrate that P. alecto TRIM5 is an active restriction factor with specificity for gammaretroviruses but not primate or nonprimate lentiviruses. TRIM21, TRIM22, and TRIM34 likely do not constitute a block to lentiviral infection in P. alecto.

### HIV-1 capsid interactions with host proteins influence susceptibility of P. alecto cells to infection.

**FIG 5:**
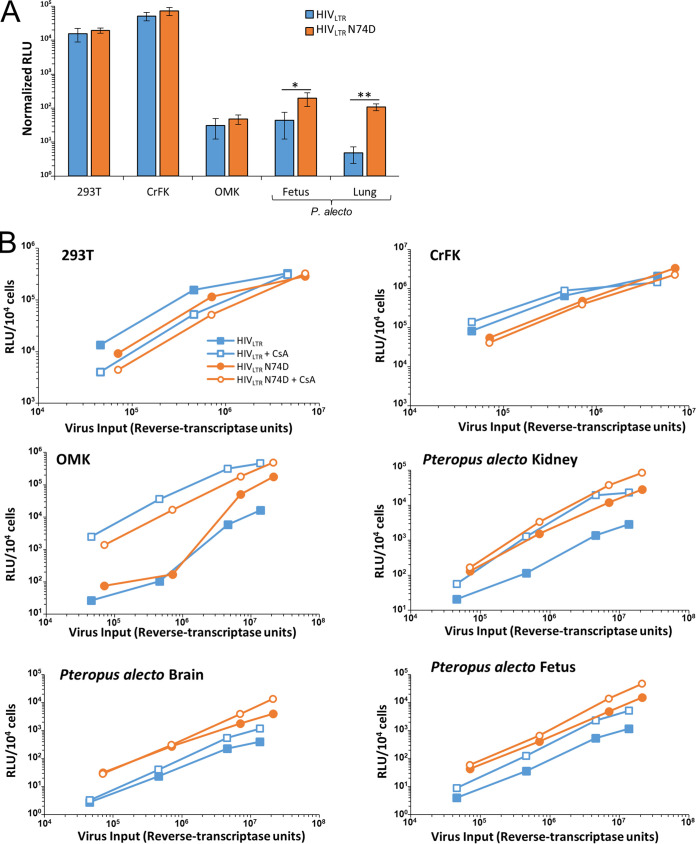
Cumulative effects of N74D capsid mutation with CsA treatment of pteropid cells. (A) Cells were infected with luciferase-encoding HIV-1_LTR_ vectors with either a wild-type or N74D capsid, and 4 days postinfection, cells were counted and lysed and cell lysates were analyzed for luminescence. Relative light unit (RLU) data were normalized to the number of live cells. Error bars represent standard deviations of luminescence calculated in triplicate. The experiment was repeated three times, and one representative figure is shown. (B) Cells were infected and analyzed as described above in the presence of 5 μM CsA or DMSO. *, *P* < 0.05; **, *P* < 0.01.

We further characterized the effects of HIV-1 capsid mutations on infectivity in P. alecto cells by examining effects of the N74D mutant, which is known to alter HIV-1 host nuclear transport and pore protein interactions, interfere with binding of CPSF6, and reduce sensitivity to MX2 restriction (40, 60). Although the N74D mutant displayed markedly impaired HIV-1 infectivity overall in the bat cell lines versus the nonbat cell lines (Fig. 5A, orange bars), this mutation resulted in substantial increases in reporter virus luciferase expression versus WT vector in P. alecto cells (4.5-fold and 13.0-fold increases in the fetal and lung lines, respectively; Fig. 5A). To determine whether the effects of N74D and CypA binding were altering the same cellular block to infectivity or whether they were signatures of mechanistically separate impairments, we combined the N74D mutation with CsA treatment in our various cell lines (Fig. 5B). In P. alecto cells, the N74D mutation resulted in a consistent increase in virally encoded luciferase expression over a wide range of viral inputs compared to the vector harboring a wild-type capsid (Fig. 5B, closed squares versus open circles). Surprisingly, however, in CsA-treated cells, N74D augmented infection of P. alecto brain and fetus cells (open circles and squares, Fig. 5B). Cumulatively, these results imply either that the N74D mutation and CypA binding affect different aspects of the HIV-1 life cycle or that neither is sufficient to completely resolve the block to infectivity observed in P. alecto cells.

### HIV-1 is blocked at nuclear import in pteropid bat cells.

**FIG 6:**
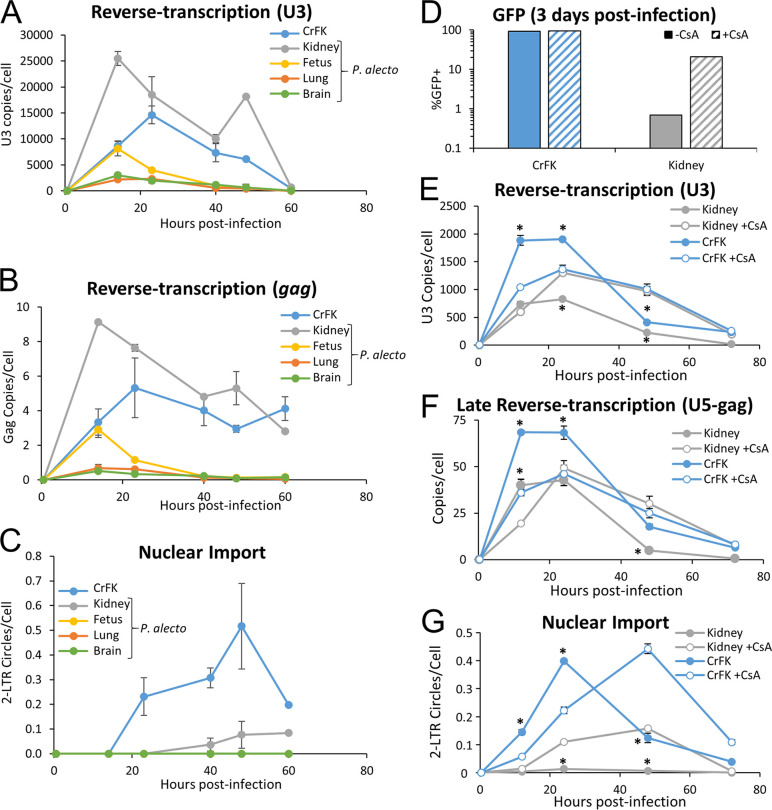
HIV-1 reverse transcription and nuclear import are impaired in Pteropus alecto cells. Cells were infected with HIV-1_LTR_ vector encoding eGFP, and total DNA was collected at the indicated time points. Viral DNA was quantified using primers specific for U3 (A), Gag (B), or 2-LTR circles (C), and the data were normalized to GAPDH. In panel C, the P. alecto fetus and lung cell curves shown are indistinguishable from (and hence obscured by) the P. alecto brain cell curve. (D to G) Cell infections were repeated with 5 μM CsA or DMSO added 1 h prior to infection and washed off after 16 h postinfection (D to G), and time courses of viral DNA species accumulation were determined (E to G). (D) At 4 days postinfection, cells were quantified for eGFP expression by fluorescence-activated cell sorter (FACS) analysis to determine viral infectivity. (E to G) Total DNA collected at indicated times postinfection from the same cell pool as that used for the experiments whose results are presented in panel D was used to determine DNA abundance for each indicated form relative to GAPDH. (E) Reverse transcription (U3 copies per cell). (F) Late reverse transcription (U5-gag copies per cell). (G) Nuclear import (copies of 2-LTR circles per cell). Error bars represent standard deviations of results from the triplicate technical replicates. Asterisks (*) indicate statistical significance (*P* < 0.01) of results of comparisons between the DMSO-treated and CsA-treated wells for a given cell type at the indicated time.

To identify where in the viral life cycle the restriction to primate lentiviruses occurs, pteropid bat cells and nonrestricting CrFK cells were infected with an HIV-1 reporter virus and stage-specific reverse-transcription products were analyzed at different times postinfection by quantitative PCR (qPCR). While HIV-1 reverse transcription was found to proceed normally in P. alecto kidney cells, with similar levels of U3 and *gag* viral DNA observed compared to CrFK cells (Fig. 6A and B), the levels of these DNAs were reduced in the P. alecto fetus, lung, and brain cell lines. Levels of 2-LTR circles, representing a dead-end form of the viral DNA that forms in the nucleus only after nuclear import of the viral preintegration complex, were significantly reduced in all P. alecto cell lines compared to control cells (Fig. 6C), even kidney cells, demonstrating impairment of nuclear import. To gain insight into how CsA relieves the block to primate lentiviral infection in pteropid cells, we repeated the measurement of viral cDNA products in cells treated with 5 μM CsA or dimethyl sulfoxide (DMSO) prior to infection. Quantification of eGFP expression in cells at 4 days postinfection confirmed again that CsA improved the infectivity of the pteropid cells under these conditions, with a 30.3-fold increase in eGFP-expressing cells observed (Fig. 6D). In CrFK cells, CsA treatment delayed accumulation of U3 and U5-gag DNAs and the 2-LTR nuclear import marker DNA mildly, with an approximately one-third reduction in the levels of these products observed up to 24 h, which converted to an increase compared to the DMSO control at 48 h (Fig. 6E to G). In contrast, accumulation of U3 and U5-gag DNA in the bat cells was mildly increased by CsA treatment at both 24 and 48 h, and levels of 2-LTR circles were markedly increased from a minimal level at both time points (Fig. 6E to G). To examine the processivity of reverse transcription under these conditions, we also used primers specific for the minus-strand DNA that forms following the second-strand transfer; these late-forming reverse-transcription products showed kinetics and relative abundances between cell lines and conditions similar to the levels seen with the total reverse-transcription products (Fig. 6F). This indicates that the variable impairment observed in total reverse-transcription products in P. alecto cells (Fig. 6A and E) was most likely occurring at or before initiation of reverse transcription and that the processivity of reverse transcriptase in P. alecto cells was not substantially altered. Strikingly, 2-LTR circle formation was significantly enhanced by CsA treatment in the P. alecto kidney cells, with a peak level of formation at 48 h that was 22.6-fold higher than that seen with the DMSO-treated controls (Fig. 6G). These results demonstrate that the primary effect of CsA on enhancement of HIV-1 infectivity occurs after initiation of reverse transcription at or before nuclear import.

### MX2 depletion partially increases HIV-1 infection and is synergistic with inhibition of CypA binding.

The data accumulated thus far in this study suggested the possible involvement of the host factor MX2, a recently described retroviral restriction factor. MX2 acts early postentry, depends on CypA interaction with the viral particle, and instigates a block to viral infectivity at or before nuclear import (40–42). P. alecto encodes an MX2 homolog with 72% identity to that of the human protein, and we confirmed this sequence by cloning and sequencing the entire open reading frame from P. alecto cDNA (Fig. S5). *A priori*, the activity of P. alecto MX2 against HIV-1 was not predictable from aligning the P. alecto protein with human, ovine, and canine proteins. The capsid binding domain of human MX2 is located in the N-terminal 25 amino acids (40, 61–63). This domain also contains a potential nuclear localization signal (NLS) (62, 64). Deletion of the N-terminal 25 amino acids eliminates the ability of MX2 to block HIV-1 infection (40, 62, 65). Moreover, a triple-arginine motif in the N-terminal 25 amino acids is required (63, 66). Accordingly, while several primate MX2 proteins are active against HIV-1, two nonprimate MX2 proteins (ovine and canine) are not (43). As is clear from Fig. S5, the P. alecto N-terminal 25 amino acids diverge substantially from human, ovine, and canine MX2, and the RRR motif is absent.

10.1128/mBio.01854-20.5 FIG S5 MX2 multiple-sequence alignment. A multiple-sequence alignment of MX2 proteins was generated in Clustal Omega. MX2 from sequenced P. alecto cDNA was used, while NCBI-deposited sequences were used for Homo sapiens (NM_002463.2), Canis lupus familiaris (NM_001003133.1), and Ovis aries (NM_001078652.1). Yellow highlighting indicates sequenced cDNA P. alecto amino acid differences from NCBI reference sequence XP_006916792.1. The red line delineates the HIV-1 capsid binding region. Download FIG S5, TIF file, 2.4 MB. Copyright © 2020 Morrison et al. 2020 Morrison et al. This content is distributed under the terms of the Creative Commons Attribution 4.0 International license.

**FIG 7:**
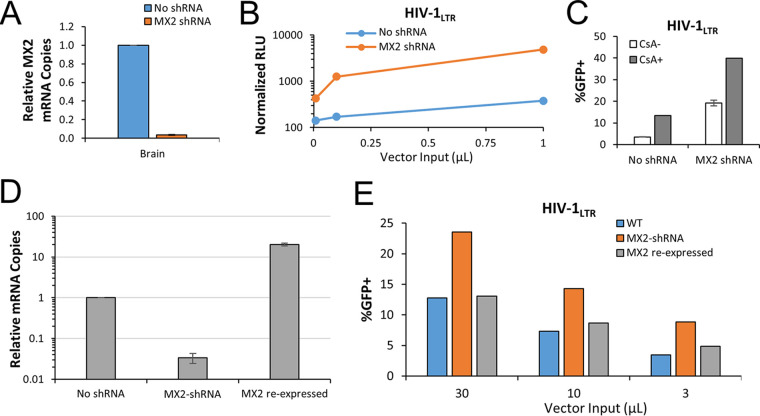
Depletion of MX2 partially relieves blocks to HIV-1 infectivity. P. alecto kidney, fetus, and brain cells were transduced with lentiviral vectors encoding mCherry and an shRNA cassette specific for bat *MX2*. (A) Stably transduced cells were analyzed for *MX2* transcript expression by qPCR normalized to GAPDH. (B) Wild-type and *MX2* knockdown brain cells were challenged with a luciferase-encoding HIV-1_LTR_ vector, and 3 days postinfection, cells were counted, lysed, and analyzed for luminescence. RLU data were normalized to the number of healthy cells and are shown as average RLU reading ± standard deviation. (C) Wild-type and *MX2* knockdown brain cells were challenged with an eGFP-encoding HIV-1_LTR_ vector in the presence of DMSO (CsA-) or 5 μM CsA (CsA+). Cells were cultured for 48 h before GFP was quantified by flow cytometry. (D) MX2 knockdown brain cells were transduced with lentiviral vector encoding a P. alecto MX2 cDNA and a puromycin selection cassette (“back-complement” cells). Puro stable cells were isolated, and *MX2* transcripts were quantified as described above. (E) Wild-type, MX2 knockdown, and recomplemented cells were challenged with eGFP-encoding HIV-1_LTR_ and analyzed as described for panel C. The data shown are representative of results from a total of three experiments performed.

To probe the potential role of P. alecto MX2, P. alecto brain cells were transduced with lentiviral vectors encoding small hairpin RNAs (shRNAs) targeting MX2 and were sorted by flow cytometry for a coencoded fluorescent marker, mCherry. This yielded an effective knockdown, with transcripts at less than four percent of control cell levels (Fig. 7A). Brain cells with and without MX2 knockdown were then infected with HIV-1, and cells were harvested for determination of virally encoded luciferase activity or eGFP expression. The MX2-depleted cells were more permissive to infection, with increases in viral reporter gene expression ranging from 3.0-fold to 12.7-fold for luciferase-encoding HIV-1_LTR_ (Fig. 7B) and a 5.5-fold increase in infectivity observed for eGFP-encoding HIV-1_LTR_ (Fig. 7C). Notably, even with MX2 transcripts at very low levels (Fig. 7A), CsA treatment still significantly improved reporter virus infectivity (Fig. 7C), showing that the effects of MX2 depletion are independent of CypA binding in bat cells. To confirm the specificity of the results, MX2 was reexpressed using a cDNA with synonymous mutations in the shRNA recognition regions. Transduced cells were selected with puromycin, and restoration of mRNA levels was confirmed (Fig. 7D). MX2 reexpression reduced the infectivity of eGFP-encoding HIV-1_LTR_ to the levels observed in wild-type cells (Fig. 7E), confirming that MX2 plays a key role in limiting HIV-1 infection in pteropid cells. MX2 cannot quantitatively account for the 1,000-fold decrease of HIV-1_LTR_ infectivity in P. alecto cells compared to human cells (Table S1), which suggests that additional antiviral factors remain to be identified in these cells.

## DISCUSSION

Autonomous cell-intrinsic restriction factors are key components of innate immune defense against viral infections. These factors have evolved the ability to directly interfere with conserved, essential elements of viral replication such as genome replication, intracellular trafficking, and protein synthesis, which in turn imposes reciprocal antagonistic selection pressures on the virus for passive evasion mechanisms or active antagonism of host restriction factors. Amplified over millennia, this selection pressure, combined with evolutionary divergence of host dependency factors, results in species-specific patterns of antiviral defense and infection, typified by the narrow tropisms of the primate lentiviruses. One in five mammalian species is a bat species, and these animals are especially intriguing in regard to evolved cell-intrinsic antiviral defenses because they are important reservoir species for a variety of viruses that are highly pathogenic in humans, such as the currently pandemic SARS-CoV-2.

Despite the significant phylogenetic distance between bats and other mammalian orders, we found that four different cell lines derived from the yinpterochiropteran bat species P. alecto are highly permissive to a wide range of retroviruses, including spumavirus (foamy virus), lentivirus (FIV, EIAV), and gammaretrovirus (NB-MLV) (Fig. 1). In contrast, we observed target cell-dependent postentry blocks to infectivity for HIV-1, SIV, and N-MLV which were consistent with either the presence of a restriction factor or the absence of a necessary dependency factor. Heterokaryons between permissive cells and P. alecto cells maintained the nonpermissive HIV-1 infection phenotype, indicating the presence of a dominant restriction factor (Fig. 2). We cannot exclude the possibility that P. alecto cells also lack dependency factors needed for efficient infection; however, additional evidence also supported the idea of the presence of specific viral restriction in the bat cells. First, the disparity in the levels of infectivity of bat cells observed for N-MLV and NB-MLV (Fig. 1) was highly suggestive of an Fv1 or TRIM5-like restriction factor, as these two viruses differ by only a single amino acid in capsid. Second, the boost in HIV-1 infectivity observed following CsA treatment or modification of the CypA binding loop (Fig. 3) suggests that the necessary HIV-1 dependency factors are present in bat cells. Finally, the P. alecto type I IFN locus mediates constitutive expression of IFN-alpha along with expression of IFN-stimulated genes such as tetherin and Mx1 genes, suggesting that bat cells may be intrinsically armed to defend against viral infections (12).

The N-MLV and NB-MLV infectivity phenotypes led us to investigate the possibility of TRIM5 restriction in pteropid cells. Pteropus alecto TRIM5 ectopic expression conferred resistance to N-MLV but not NB-MLV or diverse lentiviruses (Fig. 4C), indicating that the pteropid TRIM5 that we cloned represents a functional restriction factor. Extensive colonization of ancestral pteropid species with betaretroviruses and gammaretroviruses (25, 26) likely shaped the recognition of pteropid TRIM5 with respect to these retroviral genera. No endogenized lentiviral sequences or extant lentiviruses with tropism for bat species have been identified so far, which may suggest that the lack of P. alecto TRIM5 targeting of viruses of the *lentivirus* genus (Fig. 4F) is perhaps due to a lack of selection pressure to evolve or maintain SPRY domain interactions with the capsid proteins of lentiviruses.

Important findings in this work are that CsA markedly affects HIV-1 infectivity and yet expression of multiple bat TRIM proteins does not and that knockdown of the identified TRIM5 protein also has no effect (Fig. 3 and 4). Nevertheless, the effects of CsA treatment on HIV-1 infectivity in P. alecto cells (Fig. 3), combined with the lack of anti-HIV-1 activity observed following ectopic expression of P. alecto TRIM5, suggested that these cells might harbor a restriction factor akin to the TRIMCyp proteins of New and Old World monkeys. However, we were unable to identify TRIMCyp homologs in the P. alecto genome or transcriptome, and 5′RACE performed using P. alecto RNA and CypA gene-specific primers also did not reveal CypA fusion genes. This does not eliminate the possibility of the existence of a TRIMCyp fusion protein, but additional features indicate that such a restriction would not adhere closely to the existing mechanistic framework. Restriction of HIV-1 in P. alecto cells is not saturable, whereas TRIMCyp in OMK cells was readily abrogated by VLP saturation, as expected from many prior studies, under the same experimental conditions (Fig. 2B). Furthermore, FIV interacts with CypA and is notably sensitive to TRIMCyp restriction (Fig. 3A), but we observed that FIV (and EIAV) were not restricted in P. alecto cells (Fig. 1); CsA treatment also had no effect on FIV infectivity despite relieving the TRIMCyp-mediated block in OMK cells (Fig. 3A). If a CypA domain-containing protein is acting as an HIV-1 restrictor in P. alecto cells, then it would seem to be mechanistically distinct in binding or function from previously described TRIMCyp fusions.

Quantification of HIV-1 reverse-transcription products following infection revealed that the HIV-1 life cycle is blocked before integration in bat cells, with the most prominent deficit mapping to nuclear import (Fig. 6). Bat fetus, brain, and lung cells showed various but significant reductions in the levels of accumulation of viral cDNA compared to the nonrestricting CrFK cells, suggesting that the virus is additionally inhibited in these cells during or before reverse transcription. In the bat kidney cell line, in contrast, U3 and *gag* products accumulated to levels similar to those seen in CrFK cells whereas the levels of 2-LTR circles, a dead-end by-product of full-length viral cDNA that forms in the nucleus, were significantly reduced. This result indicates that HIV-1 is blocked at nuclear import in P. alecto kidney cells, though there may also be blocks at reverse transcription that were not further characterized in this study.

The block to HIV-1 infectivity at nuclear import, coupled with the observed CsA effects, was highly suggestive of an MX2-like restriction in the bat cells. The mechanism by which MX2 blocks HIV-1 infection in human cells has not been fully resolved, but the block maps to postentry events in the viral life cycle that culminate in integration, and the restriction appears to depend on CypA binding of the viral capsid core (40–42, 63, 66–68). In addition, CypA loop binding mutants as well as the N74D mutation can eliminate or reduce MX2 restriction (40–43), consistent with the improved HIV-1 infectivity that we observed with those capsid mutants (Fig. 3 and 5). Both CypA depletion and CsA treatment have been reported to rescue MX2 restriction of HIV (41). Passage of HIV-1 in the presence of MX2 in SupT1 cells also selected for an A88T mutation in the CypA binding loop (41). The picture is complex, as the effects of CsA are cell line dependent and were not observed in MT4 cells that ectopically express MX2, so the CypA interaction may not be necessary for MX2 restriction in all cell types (43). Under the latter conditions, viral passage selected for mutants in capsid outside the CypA binding loop.

Depletion of MX2 through the activity of stably expressed shRNA partially rescued HIV-1 infectivity in the P. alecto brain cell line (Fig. 7). Interestingly, despite a greater than 95% reduction in the levels of MX2 transcripts, a CsA phenotype was still observed in the shRNA-expressing cells, suggesting that some of the roles played by CypA, or, potentially, by another protein that binds capsid in the same location, are independent of MX2. Data representing divergence in multiple regions of P. alecto MX2 (see Fig. S5 in the supplemental material), and, in particular, in the N-terminal 25 amino acids known to be important for primate MX2 proteins (40, 43, 62, 63, 65, 66), suggest that this nonprimate MX2 protein might exert antiviral activity in a manner that differs from that seen with primate MX2 proteins. Indeed, the N-terminal domain is also poorly conserved between the P. alecto protein and the other two nonprimate MX2 proteins reported to be inactive against HIV-1 (43).

In summary, bats are reservoir species for diverse human-pathogenic viral pathogens causing severe disease and yet are less commonly reported to display disease. We infected four P. alecto cell lines with retroviruses from diverse genera to determine whether viral invasions have generated counterevolution of cell-intrinsic retroviral restrictions. P. alecto cells are readily infected with multiple retroviruses, with some notable exceptions. Pteropid TRIM5 inhibited the gammaretrovirus N-MLV in a manner that depended on one amino acid in the capsid. Most interestingly, primate lentiviruses (HIV-1, SIV_mac_) but not nonprimate lentiviruses (FIV, EIAV) are blocked at early-stage life cycle steps but not by TRIM5, and this can be relieved by inhibition of CypA binding. The antiviral factor MX2 partially accounted for this restriction, but a second, non-TRIM5 CypA-based block to HIV-1 infectivity exists. These results provide a basis for further elucidation of viral restriction in bats.

## MATERIALS AND METHODS

### Cell lines.

Human HEK 293T and HT1080 cells; Crandell feline kidney (CrFK) cells from Felis catus; ferret alveolar epithelial cells (FtAEpC) and ferret kidney cells, each from Mustella putorius *furo* (69); owl-monkey kidney cells (OMK; ATCC CRL-1556); mouse 3T3 cells; and pteropid bat cell lines derived by Crameri et al. (30) from Pteropus alecto kidney (paKiT01), brain (paBrH02), fetus (paFeSV40T), and lung (paLuT02) were maintained in Dulbecco’s modification of Eagle’s medium (DMEM) with 4.5 g/liter glucose; 10% fetal bovine serum; and 1× penicillin, streptomycin, and l-glutamine. Following transduction with Tsin IRESpuro HIV-1 vectors, P. alecto and CrFK cells were selected in 2 and 5 μg/ml puromycin, respectively.

### Viral vectors and challenges.

Single-cycle vesicular stomatitis virus G protein (VSV-G)-pseudotyped luciferase-expressing or eGFP-expressing viral vectors derived from HIV-1 NL4-3R-E (HIV-1_LTR_), feline immunodeficiency virus (FIV), and Moloney murine leukemia virus (MLV) were previously described (70, 71). Minimal HIV-1-derived vectors encoding eGFP expressed from an internal human cytomegalovirus (CMV) promoter (HIV-1_CMV_) have been previously described (70). For receptor-specific infection of heterokaryon cells, the HIV-1_CMV_ vector was employed as described above, except that a CCR5-specific HIV-1 envelope from 389F1, a transmitted founder virus (72), was used in place of VSV-G. HIV-1_CMV_ capsid mutants P90A and G89A and HIV-1_LTR_ capsid mutant N74D have also been previously described (73). Equine infectious anemia virus (EIAV) packaging and eGFP transfer plasmids (74), SIV_mac251_ vector plasmids (SIV3+) (38), and human-pathogenic foamy virus vector plasmids (75) were used as indicated. All vectors were prepared by polyethylenimine (PEI) transfection in 293T cells. Briefly, 7 × 10^6^ 293T cells were plated in a T175 flask and allowed to adhere overnight. A 22-μg volume of total DNA consisting of viral packaging, a transfer plasmid, and VSV-G prepared at a 3:3:1 ratio was added to 500 μl of Optimem media without serum. An 88-μl volume of polyethylenimine (1 μg/μl in 1× phosphate-buffered saline [PBS], pH 4.5) was added to the mix, subjected to brief vortex mixing, and incubated at room temperature for 20 min. The transfection mix was added to the cells dropwise and incubated 6 to 8 h at 37°C. The medium was then replaced with fresh DMEM followed by incubation for an additional 48 to 72 h, when the medium was removed, filtered through a 0.45-μm-pore-size filter, and centrifuged over a 20% sucrose cushion to concentrate the vector. Reverse transcriptase activity of viral vector stocks was determined using previously described methods (76). Viral challenges were performed by plating 25,000 cells in a 96-well plate and allowing them to adhere overnight, and serial dilutions of each viral vector were added. At 48 to 72 h later, the medium was removed and the cells were trypsinized and resuspended in 300 μl of DMEM and analyzed for percent eGFP-positive cells on a BD FACScan system. Alternatively, for Luciferase-expressing vectors, cells were counted and lysed 3 or 4 days postinfection, and luciferase signal was analyzed with Promega BrightGlo reagent. Cyclosporine was added to a 5 μM final concentration at the time of cell plating for appropriate experiments. For VLP saturation experiments, 15,000 cells were plated in a 96-well plate, transduced with 40 μl of concentrated HIV-1 VLPs or 40 μl of control media, and infected with HIV-1_Luc_ at a multiplicity of infection (MOI) of 0.3. Three days postinfection, cells were counted and lysed in 200 μl of PBS–0.1% Tween and 50 μl of cell lysate was used to measure luciferase content with Promega BrightGlo reagent in triplicate. Luciferase signal was then normalized to cell number. Lentiviral vectors encoding mCherry and shRNA have been previously described (76). Vectors encoding P. alecto-specific MX2 sequences were derived using the following gene-specific sequences: GGAGAAAGAGACTCGCTTATA, ATGCCTACTTCTTGGAAAC, and GCTCATCACGCACATCACTAA. MX2 shRNA vectors were prepared as described above, mixed together, and then used to infect P. alecto cells in the presence of 5 μM cyclosporine. At 24 h later, cells were washed with fresh media. mCherry-positive cells were isolated by flow cytometry on a BD FACSAria II flow cytometer. To recomplement MX2 shRNA-expressing brain cells, P. alecto and MX2 cDNAs (the method of cDNA isolation is described below) harboring at least 8 synonymous mutations in the shRNA recognition sites were generated by overlap extension PCR and inserted into TsinIRESpuro using a Thermo Fisher GeneArt seamless cloning kit. Lentiviral vectors encoding the shRNA-resistant MX2 cDNA were prepared and utilized as described above for shRNA vectors to generate stably back-complemented cells. Back-complementation was confirmed for each gene by qPCR.

### Viral life cycle analysis.

A total of 3 × 10^5^ cells were infected with eGFP-encoding HIV-1_LTR_ at an MOI of 20 (Fig. 6A to C) or 2 (Fig. 6D to F), and a Qiagen DNeasy kit was used to collect total DNA from cells at between 20 min and 60 h postinfection. HIV-1 U3 (forward [Fwd], 5′-GAACTACACACCAGGGCC-3′; reverse [Rev], 5′-CTCCGGATGCAGCTCTCG-3′), Gag (Fwd, 5′-AGCAGGAACTACTAGTACCC-3′; Rev, 5′-TTGTCTTATGTCCAGAATGC-3′), late post-second-strand transfer DNA (U5-Gag; flanks the primer binding site [77]) (Fwd, 5′-TGTGTGCCCGTCTGTTGTGTGA-3′; Rev, 5′-GAGTCCTGCGTCGAGAGAGCT-3′), or 2-LTR circle (Fwd, 5′-AGTGTGGAAAATCTCTAGCAGTAC-3′; Rev, 5′-CTCCGGATGCAGCTCTCG-3′) HIV-1 DNA, as well as control cellular GAPDH (glyceraldehyde-3-phosphate dehydrogenase) (cat Fwd, 5′-ACCACAGTCCATGCCATCAC-3′; cat Rev, 5′-TCCACCACCCGGTTGCTGTA-3′; bat Fwd, 5′-CTTTGGCATCATGGAAGGACTCAC-3′; bat Rev, 5′-GGAGGCCATGTGGACCATAAGG-3′), was quantified by real-time quantitative PCR using a Roche 480 LightCycler and Roche LCDA software. Serially diluted plasmids were used as standards. LightCycler programs included an initial denaturation step at 95°C for 10 min and a melting step after amplification (65°C to 97°C; temperature transition rate, 0.11°C/s), with 45 cycles of 95°C for 10 s, 53 to 65°C (primer specific) for 10 s, and 72°C for 15 s, at a temperature transition rate of 4.4°C/s. Samples were analyzed in triplicate, and means and standard deviations of results were calculated for each. The experiments were performed in triplicate, and data from a representative experiment are shown.

### Isolation and cloning of Pteropus alecto MX2 and TRIM cDNAs.

Total RNA from Pteropus alecto lung and brain cells was collected with a Qiagen RNeasy Plus minikit and converted to cDNA using a Roche Transcriptor first-strand cDNA synthesis kit. A cDNA with 99.6% amino acid identity to the longest predicted P. alecto MX2 isoform (NCBI accession no. XP_006916792.1) was isolated by PCR (Fwd, 5′-GCTGGTTCCCGGCTAATGCC; Rev, 5′-CACCCCTGCTTTAGCAGGAGAATTTG) and subjected to Sanger sequencing by QuintaraBio. cDNAs corresponding to P. alecto TRIM5 (ELK09387), TRIM21 (XP_006905883.2), TRIM22 (XP_024905227.1), and TRIM34 (ELK09388.1) were similarly isolated and sequenced. For 5′RACE of TRIM5 mRNA, total RNA was collected from P. alecto kidney and lung cells as described above. The 5′ segments of TRIM5 transcripts were determined using a FirstChoice RLM-RACE kit (Applied Biosciences) according to the manufacturer’s instructions. The SPRY-specific primers used were 5′-GACCTATGAAAACCCCAACACGACA (outer) and 5′-GGAGGAAGAGTCCTCAAAAGCA (inner). Nested PCR products were isolated on agarose gels and then blunt cloned using a StrataClone Blunt PCR cloning kit (Agilent). Multiple clones (total, 6 to 10) were sequenced for each cell line, and only clones containing the intact 36-bp 5′RACE adapter were included for later comparison. To verify the identity of isolated P. alecto TRIM cDNAs, multiple-sequence alignments were generated with mammalian homologs using Clustal Omega and the alignments were visually inspected in MEGA7 (78). Phylogenetic analyses were conducted in MEGA7. First, we performed maximum likelihood analyses to predict the substitution model that would best fit the data, as implemented in MEGA7. The evolutionary history was inferred by using the maximum likelihood method on the basis of the Jones-Taylor-Thornton matrix-based model (79). The tree with the highest log likelihood (−8,403.24) is shown in Fig. S4 in the supplemental material. A total of 1,000 bootstrap replicates were performed, and branches are labeled with bootstrap percentages demonstrating high support for the major clades of TRIM homologs. A discrete gamma distribution was used to model evolutionary rate differences among sites (5 categories; +G, parameter = 1.7517). The tree is drawn to scale, with branch lengths measured in the number of substitutions per site. The analysis involved 16 amino acid sequences. All positions containing gaps and missing data were eliminated. There were a total of 401 positions in the final data set. MX2 transcript levels were determined by isolating total RNA from shRNA-expressing and control cells, converting total RNA into cDNA, and measuring relative transcript abundances using the qPCR methods outlined above. The primers used for qPCR analysis of MX2 were as follows: Fwd, 5′-GTGTCGGTGGGAGACAAGGAC; Rev, 5′-CCTCGCACGAGAGCTGCTTC. Mus musculus Fv1 mRNA (NM_010244.3) and amino acids (NP_034374.2) were used to interrogate the Pteropus alecto nr/nt nucleotide collection with blastn (BLASTN 2.8.0+) and tblastn (TBLASTN 2.8.0+), respectively, using default search conditions for somewhat similar sequences.

### TRIM5 knockdown.

For each well in a 24-well plate, 12.5 pmol Mission predesigned small interfering RNA (siRNA) or Mission siRNA universal negative-control no. 1 (Sigma-Aldrich) was diluted in 125 μl Opti-MEM I medium without serum. A 7.5-μl volume of Lipofectamine RNAiMAX was added to each well and mixed gently before incubation at room temperature for 15 min. Each cell line was diluted in growth media without antibiotics to 6 × 10^5^ cells per milliliter. A 0.5-ml volume of diluted cells was added to each well containing mixed siRNA and transfection reagent to give a final siRNA concentration of 20 nM. Cells were mixed and incubated for 48 h, at which point the cells were transfected again with the same mixture of siRNA and RNAiMAX. Two hours after the second transfection, HIV_LTR_-Luc was added to each well, and after an additional 48 h, cells were counted and analyzed for luminescence. The siRNA sequences used were as follows: for TRIM5 siRNA1, UGGCAGAGGCUGAGAAUGA; for TRIM5 siRNA2, AAAUAUGGUUACUGGGUUA.

### Indirect immunofluorescence and immunoblotting.

Subcellular localization experiments were performed using previously described methods (73, 76) with the variations described below. Briefly, 1 × 10^5^ cells were plated in Lab-Tek II chamber slides, allowed to adhere overnight, fixed with 4% (wt/vol) paraformaldehyde, permeabilized with methanol, stained with 4′,6-diamidino-2-phenylindole (DAPI), and imaged by laser scanning confocal fluorescence microscopy. Cell lysates were collected in radioimmunoprecipitation assay (RIPA) buffer (150 mM NaCl, 0.5% deoxycholate, 0.1% sodium dodecyl sulfate, 1% NP-40, 150 mM Tris-HCl, pH 8.0) plus protease inhibitors (complete-Mini; Boehringer). A 25-μg volume of total protein was loaded onto 10% Mini-Protean precast polyacrylamide gels (Bio-Rad) and run at 150 V for 45 min before transfer onto a 0.45-μm-pore-size polyvinylidene difluoride (PVDF) membrane. Membranes were blocked in 10% milk before incubation with appropriate antibodies overnight in 5% milk, washed three times with Tris-buffered saline (TBS)–0.1% Tween 20, incubated for 1 h at room temperature with appropriate secondary antibodies (1:5,000 dilution), and washed another three times with TBS-Tween. Membranes were developed using Luminata Crescendo ECL substrate (EMD-Millipore) and imaged using a Bio-Rad ChemiDoc XRS+ imager. The antibodies used were rat monoclonal anti-HA (Roche) (1:1,000), mouse monoclonal anti-α-tubulin (Sigma) (1:5,000), goat anti-rat IgG horseradish peroxidase (HRP) (Santa Cruz), goat anti-mouse IgG HRP (Pierce/Thermo Scientific), goat anti-rabbit IgG HRP (Pierce/Thermo Scientific), and Alexa-594-conjugated goat anti-rat (Invitrogen).

### Statistical methods.

An unpaired Student’s *t* test with a two-tailed distribution was performed for the experiments whose results are presented in Fig. 2 and 6 for comparisons between control and treatment groups. Calculated *P* values that reached significance (see figure legends for significance cutoffs) are displayed above each group (indicated with asterisks [*]).

### Data availability.

The full-length TRIM5 transcript sequence was uploaded to GenBank (accession number MT649092).
