# Supplementary figures and images for: A Potent Postentry Restriction to Primate Lentiviruses in a Yinpterochiropteran Bat
Source: mBio. 2020 Sep 15;11(5):e01854-20. doi: 10.1128/mBio.01854-20 (PMC7492736; doi:10.1128/mBio.01854-20)

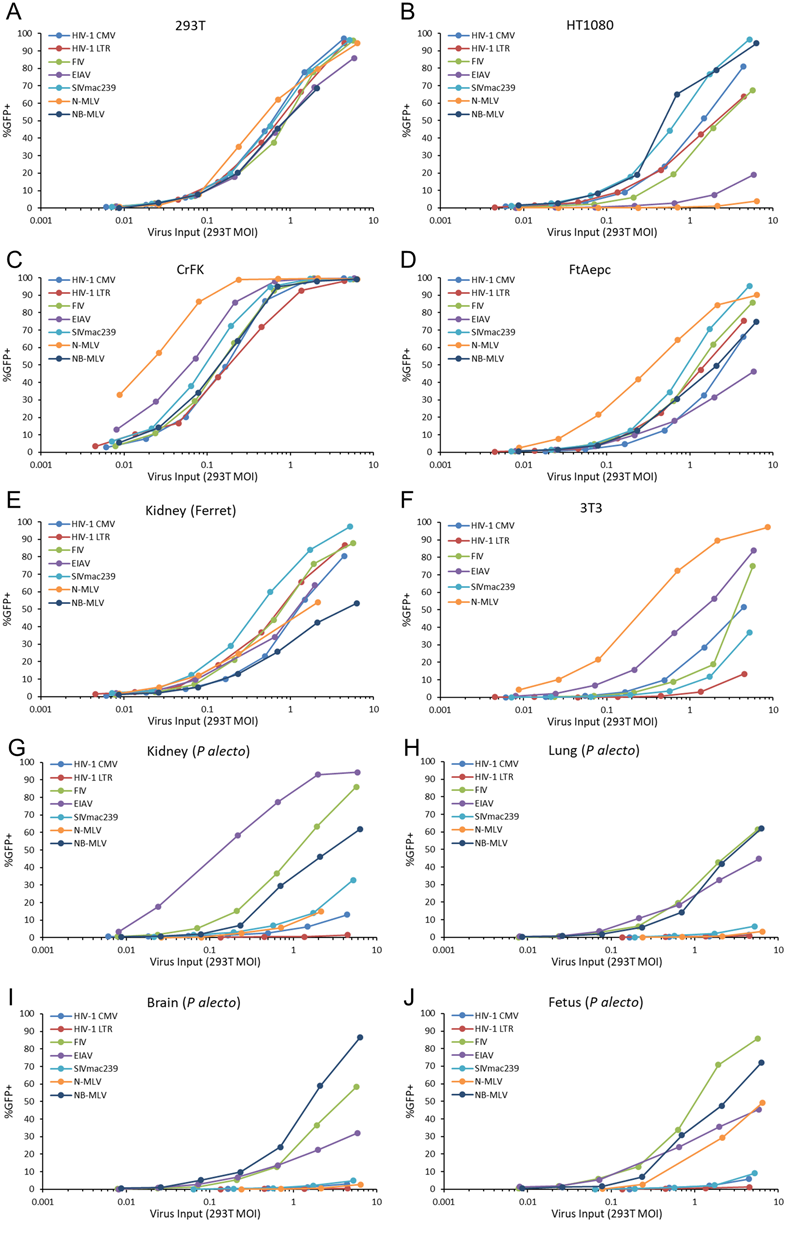

Supplement: FIG S1 [file mBio.01854-20-sf001.tif]

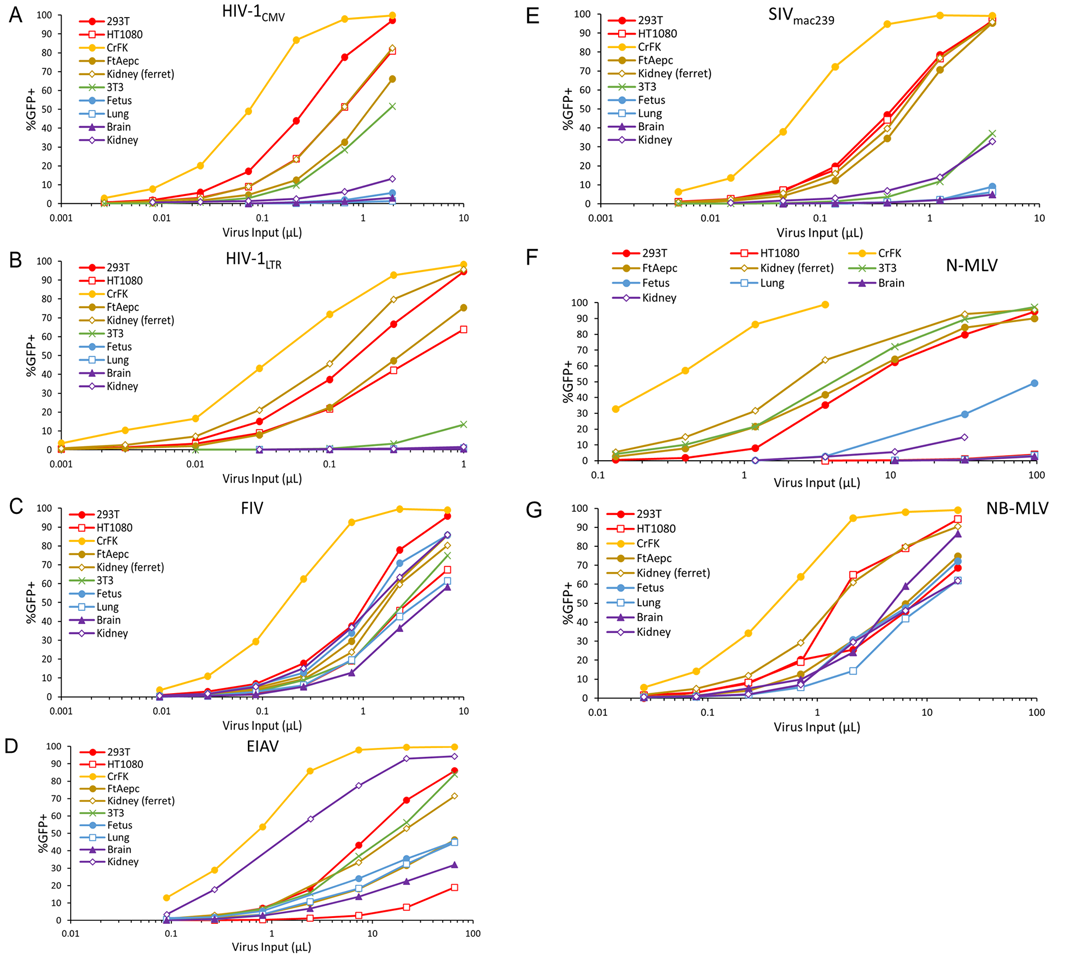

Supplement: FIG S2 [file mBio.01854-20-sf002.tif]

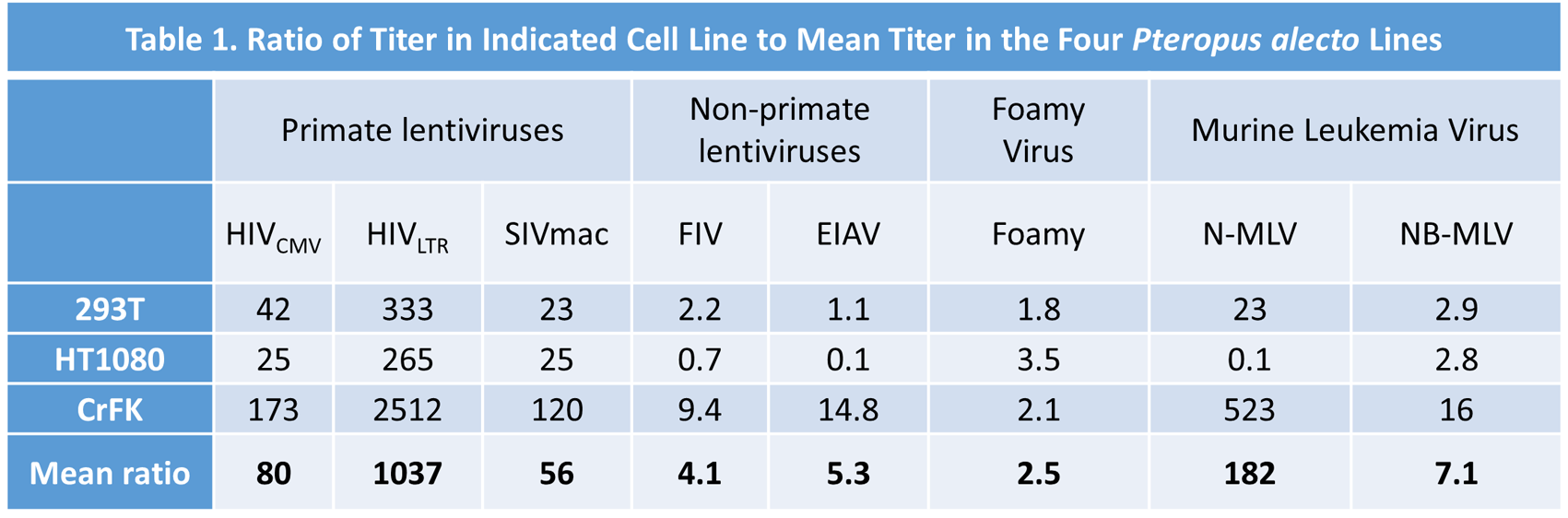

Supplement: TABLE S1 [file mBio.01854-20-st001.tif]

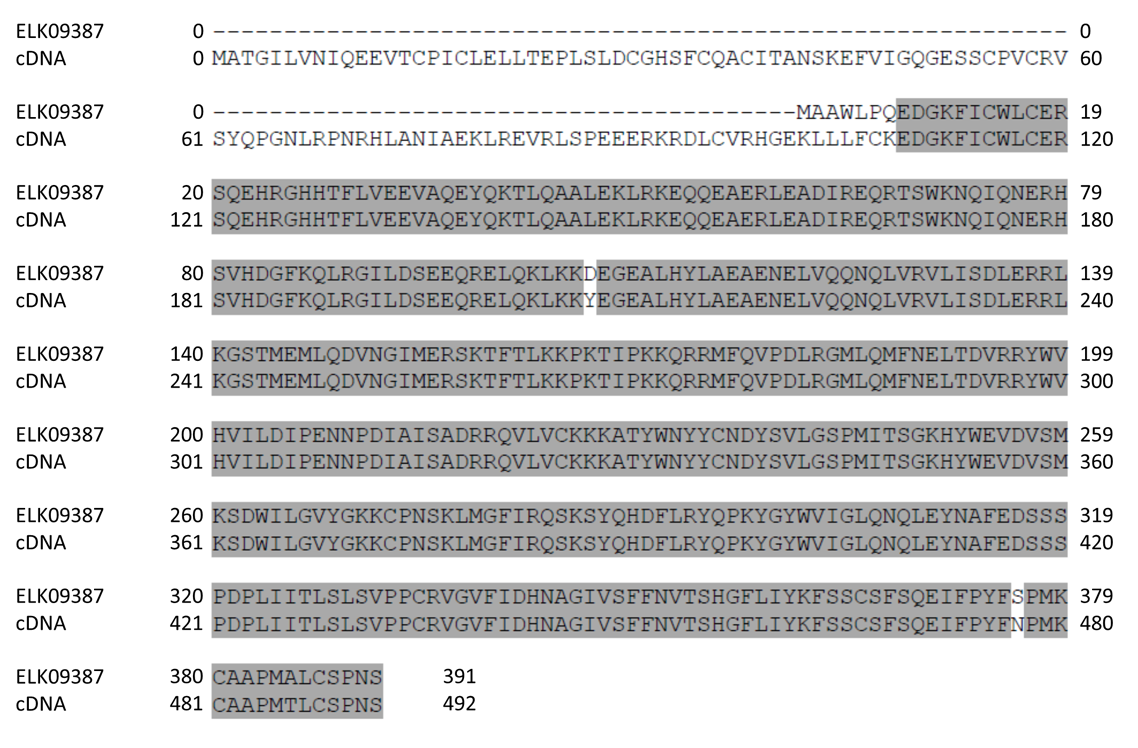

Supplement: FIG S3 [file mBio.01854-20-sf003.tif]

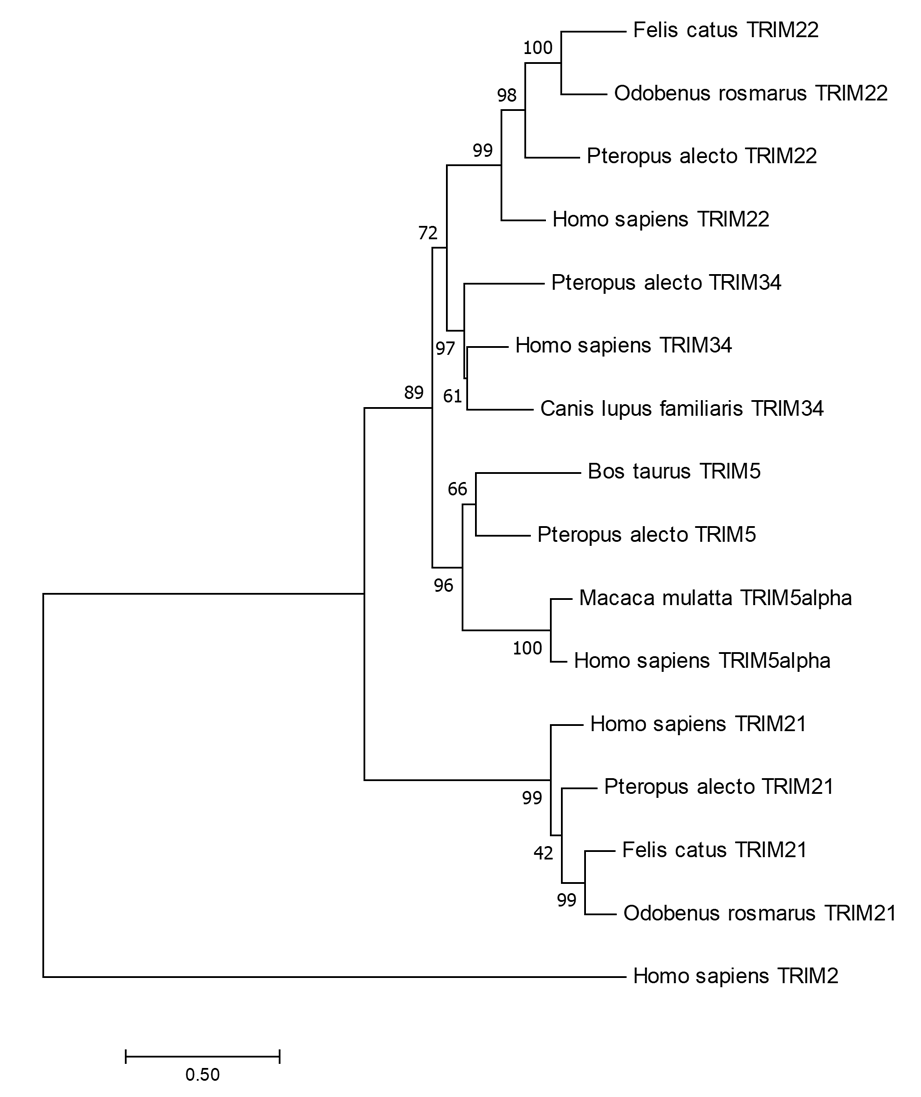

Supplement: FIG S4 [file mBio.01854-20-sf004.tif]

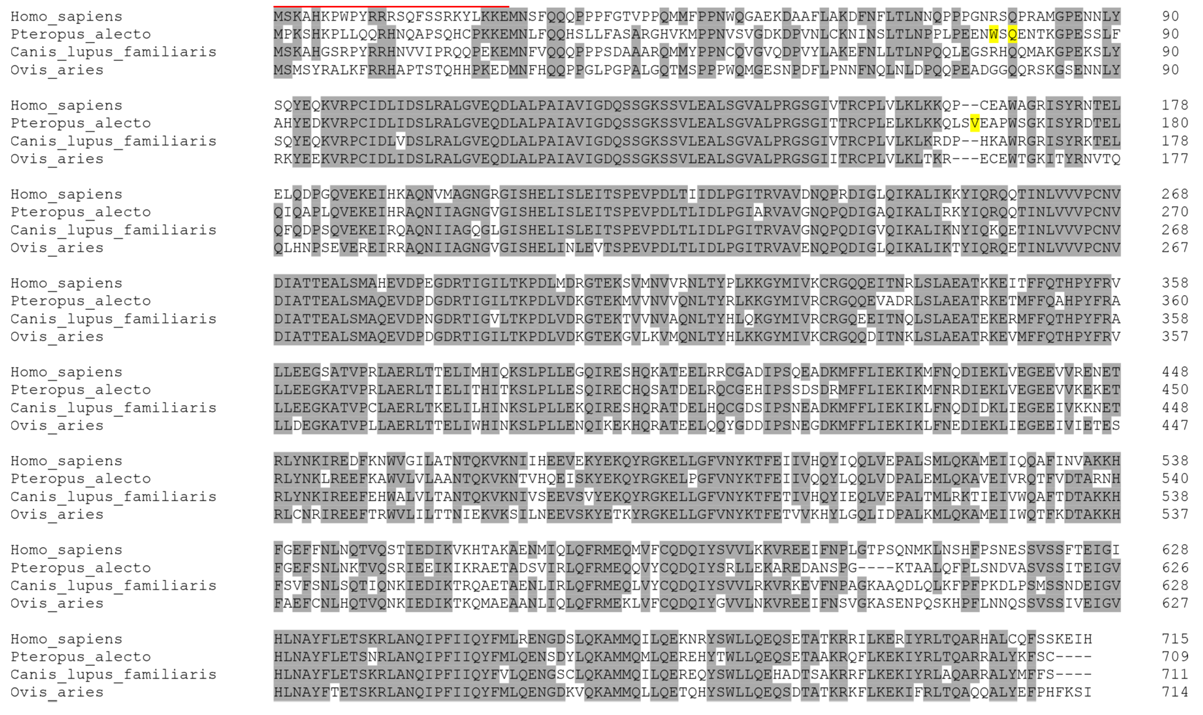

Supplement: FIG S5 [file mBio.01854-20-sf005.tif]
